# Unexpected abnormal positive pressure due to misconnection of excess gas tube

**DOI:** 10.1186/s40981-023-00677-x

**Published:** 2023-12-01

**Authors:** Atsuhiro Kitaura, Hiroatsu Sakamoto, Kensuke Toho, Shota Tsukimoto, Haruyuki Yuasa, Yasufumi Nakajima

**Affiliations:** 1 Department of Anesthesiology, Kindai University Facility of Medicine, Osakasayama, Japan; 2 Department of Anesthesiology, Kanagawa Dental University, Yokosuka, Japan

Letter to Editor

We present a case of unexpected positive pressure due to the misconnection of excess gas tubing. This report underscores the importance of performing a comprehensive pre-anesthesia check of equipment to ensure patient safety.

A 69-year-old male with ASA-PS II was scheduled for thoracoscopic esophagectomy. Initial inspection of the anesthesia machine (Apollo Draeger) on setup was performed using an automatic check. General anesthesia was induced with propofol, remifentanil, and rocuronium. After tracheal intubation, the patient was connected to a breathing circuit and mechanical ventilation started. Fresh gas flow was set at 2 L/min (1 L/min of oxygen and 1 L/min of air). Shortly after initiating ventilation (pressure control 15 cmH_2_0, rate: 12/min, PEEP 5 cmH_2_O), auto PEEP increased uncontrollably. The consultant anesthesiologist identified the issue and inspected the machine. It was found that the excess gas disposal tube was wrongly connected to the safety valve of the Jackson Ree’s circuit (Fig. 1).

**Fig. 1:**
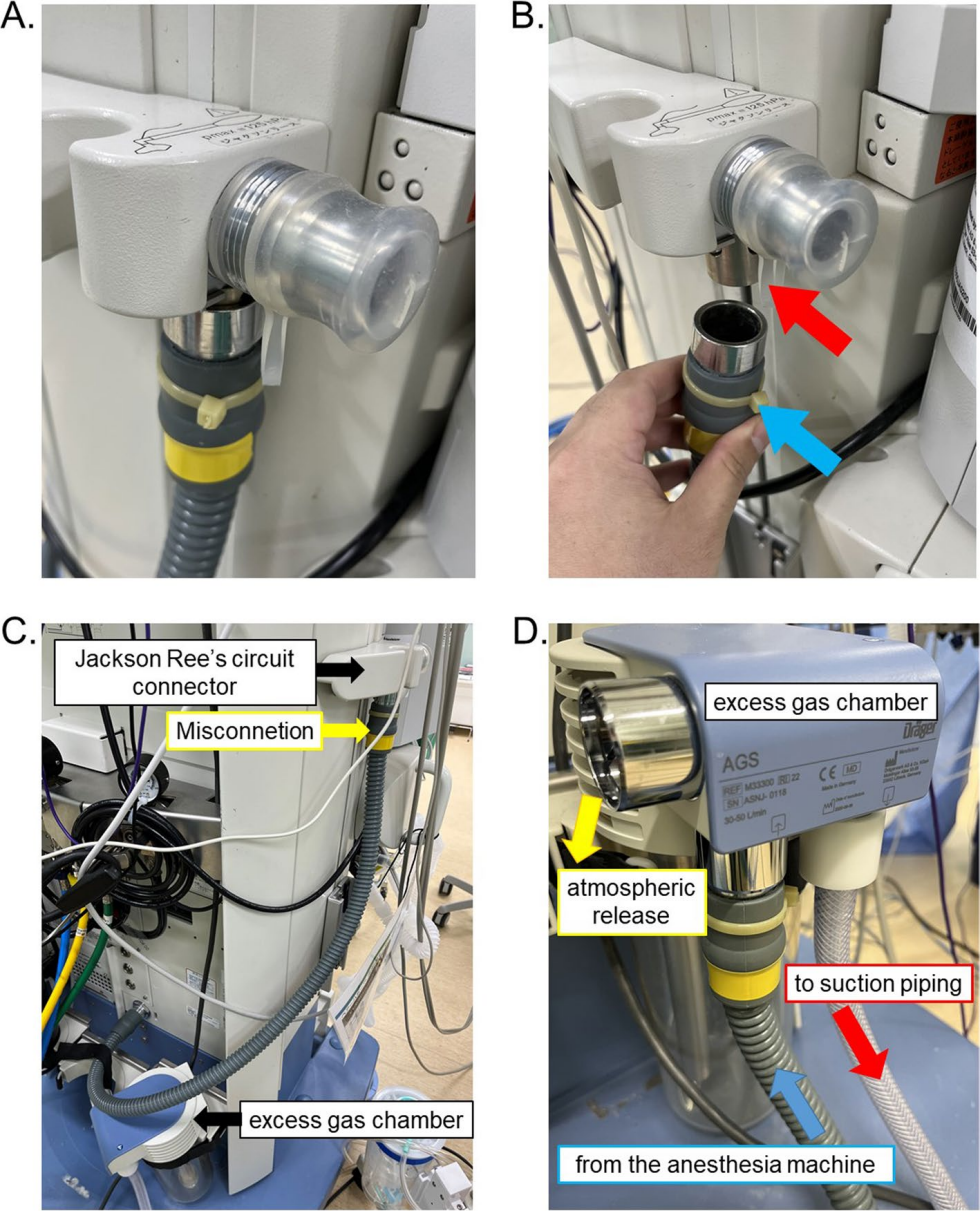
Excess gas tube misconnection to the safety valve of Jackson Ree’s system connecter. **A**–**C** Misconnection of the excess gas tube to the safety valve of Jackson Ree’s system connecter. **B** The excess gas tube (blue arrow) is misconnected to the safety valve at Jackson Ree’s circuit connecter (red arrow) on the left side of the anesthesia machine (Apollo, Draeger, Germany). Note the standard diameter of the excess gas piping with no misconnection prevention mechanisms. **D** Correct connection of the excess gas system. The excess gas tube (blue arrow) is connected to the excess gas chamber

The automatic system check includes an item for excess gas. It was performed and passed preoperatively. However, the check for excess gas involves a mechanism that uses a sensor to check the positional relationship between the ventilator system and the excess gas outlet inside the machine but does not check the exhaust. Thus, confirming that the excess gas piping is properly installed is included in the items to be manually checked before the automatic system check. A scheme explaining the structure of the anesthesia machine in this case is shown in Fig. 2. If the excess gas tube connecting the breathing circuit to the chamber is blocked, the circuit becomes a closed system. This leads to increasing pressure, ultimately compromising ventilation (Additional file 1). If a steady pressure of 30 mmHg or more is applied for more than 10 s, the safety vent is released and degassed to prevent the lungs from being continually subjected to abnormally high pressures. However, impaired venous return due to abnormally sustained high airway pressure may cause severe hypotension. If the underlying problem remains unresolved after safety vent activation, the same phenomenon may be repeated in a short period of time, resulting in impaired ventilation and potentially life-threatening effects.

**Fig. 2:**
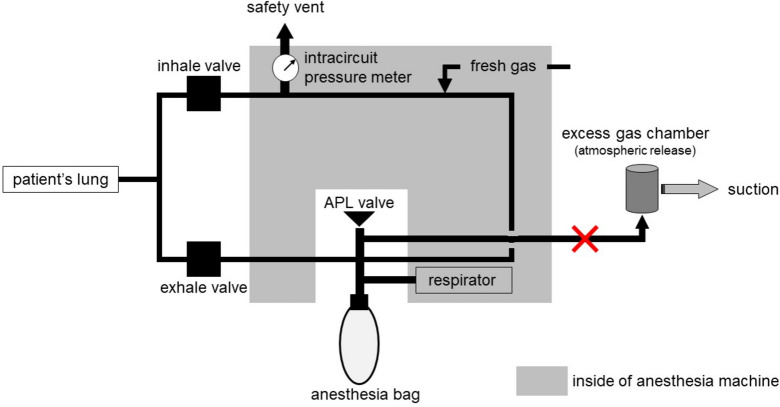
Breathing circuit in the anesthesia machine. The anesthesia machine (Apollo, Draeger) has an external excess gas system. The excess gas flow was blocked (X), at the safety valve at the Jackson Ree’s system connector in Fig. 1. APL: adjustable pressure limiting

Not only excess gas piping but also external piping for anesthesia machines can fall off. As the diameter of the excess gas piping is a standard diameter and there is no misconnection prevention mechanism, there is a risk of misconnection by staff unfamiliar with the machine structure. At our facility, there was another case of misconnection to the joint of general-purpose mounts (Nihon Kohden Co., Fig. 3). It is important for anesthesiologists to check that the excess gas system is in place before initiating ventilation [1]. In the future, the excess gas system will be built into the anesthesia machine, which should reduce the number of similar problems. As many institutions will continue to use conventional anesthesia machines alongside these new machines, the potential for misconnection requires attention.

**Fig. 3:**
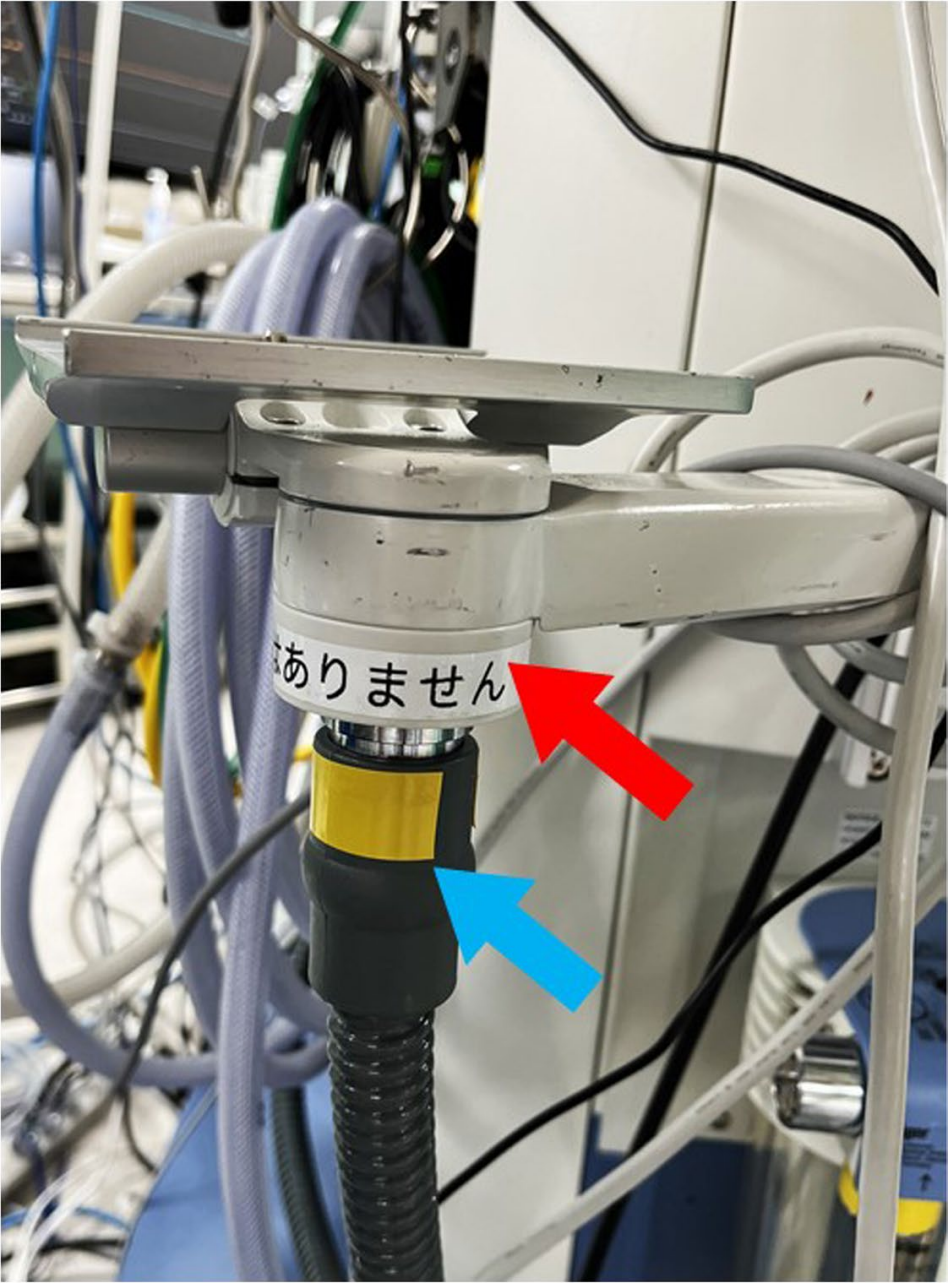
Misconnection of the excess gas tube to a mount equipment on the anesthesia machine in another case. The excess gas tube (blue arrow) is misconnected to the safety valve of Jackson Ree’s system on the mount equipment (red arrow, Nihon Kohden Co.) attached to the left side of the anesthesia machine (Apollo, Draeger, Germany)

### Supplementary Information

**Additional file 1. **Video of the reproduction experiment. The anesthesia machine (Apollo, Draeger, Germany) is prepared as normal except for the excess gas tube misconnection to the safety valve of Jackson Ree’s system connecter. A test lung is attached to the semi-close breathing circuit, an adjustable pressure limiting valve is fully opened, fresh gas is set at 2 L/min, and the ventilator is operated in pressure control management mode. Auto PEEP appears and gradually increases.

## Data Availability

Not applicable.

## Abbreviation

PEEP: Positive end-expiratory pressure
